# Robotic Versus Open Pancreatoduodenectomy With Vein Resection and Reconstruction: A Propensity Score-Matched Analysis

**DOI:** 10.1097/AS9.0000000000000409

**Published:** 2024-03-26

**Authors:** Niccolò Napoli, Emanuele Federico Kauffmann, Michael Ginesini, Armando Di Dato, Virginia Viti, Cesare Gianfaldoni, Lucrezia Lami, Carla Cappelli, Maria Isabella Rotondo, Daniela Campani, Gabriella Amorese, Caterina Vivaldi, Silvia Cesario, Laura Bernardini, Enrico Vasile, Fabio Vistoli, Ugo Boggi

**Affiliations:** * From the Division of General and Transplant Surgery, University of Pisa, Pisa, Italy; † Division of Radiology, Azienda Ospedaliero Universitaria Pisana, Pisa, Italy; ‡ Division of Pathology, University of Pisa, Pisa, Italy; § Division of Anesthesia and Intensive Care, Azienda Ospedaliero Universitaria Pisana, Pisa, Italy; ‖ Department of Translational Research and New Technologies in Medicine and Surgery, University of Pisa, Pisa, Italy; ¶ Division of Medical Oncology 2, Azienda Ospedaliero Universitaria Pisana, Pisa, Italy.

**Keywords:** pancreatoduodenectomy with vascular resection, propensity scored matched comparison, robotic pancreatoduodenectomy with vascular resection, robotic pancreatoduodenectomy with vein resection

## Abstract

**Objective:**

This study aimed to compare robotic pancreatoduodenectomy with vein resection (PD-VR) based on the incidence of severe postoperative complications (SPC).

**Background:**

Robotic pancreatoduodenectomy has been gaining momentum in recent years. Vein resection is frequently required in this operation, but no study has compared robotic and open PD-VR using a matched analysis.

**Methods:**

This was an intention-to-treat study designed to demonstrate the noninferiority of robotic to open PD-VR (2011–2021) based on SPC. To achieve a power of 80% (noninferiority margin:10%; α error: 0.05; ß error: 0.20), a 1:1 propensity score-matched analysis required 35 pairs.

**Results:**

Of the 151 patients with PD-VR (open = 115, robotic = 36), 35 procedures per group were compared. Elective conversion to open surgery was required in 1 patient with robotic PD-VR (2.9%). One patient in both groups experienced partial vein thrombosis. SPC occurred in 7 (20.0%) and 6 patients (17.1%) in the robotic and open PD-VR groups, respectively (*P* = 0.759; OR: 1.21 [0.36–4.04]). Three patients died after robotic PD-VR (8.6%) and none died after open PD-VR (*P* = 0.239). Robotic PD-VR was associated with longer operative time (611.1 ± 13.9 minutes vs 529.0 ± 13.0 minutes; *P* < 0.0001), more type 2 vein resection (28.6% vs 5.7%; *P* = 0.0234) and less type 3 vein resection (31.4% vs 71.4%; *P* = 0.0008), longer vein occlusion time (30 [25.3–78.3] minutes vs 15 [8–19.5] minutes; *P* = 0.0098), less blood loss (450 [200–750] mL vs 733 [500–1070.3] mL; *P* = 0.0075), and fewer blood transfusions (intraoperative: 14.3% vs 48.6%; *P* = 0.0041) (perioperative: 14.3% vs 60.0%; *P* = 0.0001).

**Conclusions:**

In this study, robotic PD-VR was noninferior to open PD-VR for SPC. Robotic and open PD-VR need to be compared in randomized controlled trials.

## Abstract 2

Mini-abstract:

In a 1:1 propensity score-matched analysis, robotic pancreatoduodenectomy with vein resection (PD-VR) was noninferior to open PD-VR with respect to the incidence of severe postoperative complications. In addition, robotic surgery is associated with less blood loss, fewer blood transfusions, and equivalent R0 resection rates.

## INTRODUCTION

After neoadjuvant treatment, pancreatoduodenectomy (PD) with vein resection and reconstruction (PD-VR) is currently the standard of care for patients with borderline resectable pancreatic ductal adenocarcinoma (PDAC).^1^ According to the National Comprehensive Cancer Network guidelines, PDAC located in the head/uncinate process of the pancreas is considered borderline resectable in the presence of tumor contact with the superior mesenteric/portal vein >180°, contact of ≤180° with contour irregularity of the vein, or thrombosis of the vein when vascular reconstruction is still possible. Other anatomic factors demonstrating borderline resectability include tumor contact with the inferior vena cava, common hepatic artery, superior mesenteric artery (≤180°), and variant arterial anatomy. Tumors showing only marginal contact with the superior mesenteric/portal vein are anatomically resectable and may not receive neoadjuvant treatments.^2^ However, some patients may require PD-VR.

Therefore, neoadjuvant therapy is necessary for borderline resectable PDAC. Afterward, if post-treatment resectability criteria are met, there is a high probability that vein resection will be required. On the other hand, in resectable PDAC, neoadjuvant treatments are optional; however, vein resection may unexpectedly become necessary if vascular abutment is incidentally discovered during surgery. In both cases, the aim of vein resection is to increase the probability of radical tumor resection (R0). In practical terms, PD-VR can be either planned (40%) or unplanned (60%)^3^ and is required in 6 to 65% (average 18%) of patients undergoing PD.^4^ Although most patients should receive neoadjuvant treatments, benchmark data on PD-VR demonstrate that this occurs in only approximately one-third of patients.^5^

Robotic PD has been gaining momentum. Following selective implementation,^6^ further development asks the difficult question of safety and oncologic adequacy of PD-VR. Both Miami and Brescia guidelines on minimally invasive pancreatic resections acknowledged the lack of specific studies and recommended that minimally invasive PD-VR should only be performed by highly experienced surgeons working in high-volume centers, and that outcomes should be recorded in prospective registries and/or databases.^6,7^ Robotic assistance improves surgical dexterity in minimally invasive procedures and could facilitate the safe implementation of minimally invasive PD-VR.^8–12^ However, data are sparse, and sound comparisons with open PD-VR are lacking.

Our group pioneered open PD-VR, with the first procedures performed more than 40 years ago. We reported on open PD-VR,^13^ robotic PD-VR,^12,14^ pancreatectomy with arterial resection,^15,16^ pancreatectomy with resection of the superior mesenteric artery,^17^ and robotic PD with arterial resection.^18^ Herein, we provide a propensity score-matched analysis of robotic versus open PD-VR.

## METHODS

The data of patients who underwent PD-VR between May 2011 and December 2021 at the Division of General and Transplant Surgery of the University of Pisa were extracted from a prospective database for retrospective analysis.

This study was approved by the Institutional Ethical Board of the University of Pisa (PSM-rPD 1.0—April 12, 2023) and was performed according to the principles of the Declaration of Helsinki and the Strengthening the Reporting of Observational Studies in Epidemiology guidelines on reporting on observational studies.^19,20^ The Ethical Board of the University of Pisa waived the need for informed consent due to the observational nature of this study.

Surgical techniques for either open or robotic PD-VR have been previously reported.^12–14^ At our Institution, all patients undergoing open or robotic PD were admitted overnight to the intensive care unit.

### Study Design

This intention-to-treat study was designed to demonstrate the noninferiority of robotic to open PD-VR based on the incidence of severe postoperative complications (SPC). The secondary study endpoints were postoperative mortality, functional recovery, textbook outcome, length of hospital stay, clinically relevant postoperative pancreatic fistula (POPF), grade C POPF, delayed gastric emptying (DGE) (grades B and C), postpancreatectomy hemorrhage (PPH) (grades B and C), repeat surgery, and hospital readmission. In patients with PDAC, additional secondary study endpoints were R0 resection and number of examined lymph nodes.

Robotic and open PD-VR groups were compared following 1:1 propensity score matching to minimize treatment selection bias. The following matching variables were considered: age, body mass index, American Society of Anesthesiologists (ASA) score, diabetes, heart disease, chronic pulmonary disease, and administration of neoadjuvant therapy.

All robotic PD-VR in this study were performed upon completion of the learning curve in standard robotic PD.^21^

### Sample Size Calculation and Statistical Power of the Study

Considering a discharge rate of SPC of 28% for both groups, based on the benchmark outcomes for open PD-VR,^5^ the study had a power of 80% considering a noninferiority margin of 10%, an α error of 0.05, and a β error of 0.20. Therefore, to demonstrate the lack of difference between the standard (ie, open PD-VR) and experimental (ie, robotic PD-VR) operations, 35 patients per group were required to be 80% certain that the upper limit of a 1-sided 95% confidence interval—or equivalently a 90% 2-sided confidence interval—would exclude a difference in favor of the standard procedure exceeding 10%.^22^

### Definition of Main Outcome Measures

Pancreas-specific complications were defined according to the International Study Group of Pancreatic Surgery (ie, POPF, DGE, PPH, and chyle leak).^23–26^ Grades B and C POPF were considered clinically relevant. Bile leaks were defined according to the International Study Group for Liver Surgery.^27^

The Clavien–Dindo classification was used to define and grade postoperative complications. Severe complications were graded as grade ≥3a. The highest grade was considered.^28^

Textbook outcome was defined according to van Roessel et al^29^ as absence of POPF, bile leak, PPH (all grade B/C), SPC, 30-day readmission, and 30-day or in-hospital mortality.

Functional recovery was defined as reported by van Hilst et al^30^ in the LEOPARD-2 trial (ie, when meeting all the following criteria: adequate pain control with only oral analgesia, independent mobility, ability to maintain more than 50% of the daily required caloric intake, no need for intravenous fluid administration, and no signs of infection temperature <38.5°C).

Postoperative mortality was defined as death within 90 days after surgery or during hospital stay.

Resection margins were considered negative (R0) when no tumor cells were found ≤1 mm from the margins.^12^

### Patient Selection for PD-VR

Indications for surgery were established by a multidisciplinary tumor board. As previously described, we prefer an open approach in patients with borderline resectable tumors, such as those with vein narrowing or thrombosis, and in those with tumor contact with the superior mesenteric artery.^12,14,31^ After some experience, tumors showing “less invasive” features, but still potentially requiring PD-VR, were carefully considered for a robotic approach. Some of these patients underwent open PD-VR when the robot was not available within 4 weeks.^32^ In addition, when the tumor abutment of the superior mesenteric/portal vein was incidentally discovered during robotic PD, the procedure was completed robotically if vein resection and reconstruction could be safely performed.

Patients requiring arterial resection were primarily managed using an open approach.^16^ For the purpose of this analysis, all patients who underwent arterial resection with robotic assistance were excluded.

### Anticoagulation Policy

Anticoagulation did not increase over the standard protocol for the prevention of deep venous thrombosis, which is based on low molecular weight heparin. Antithrombotic prophylaxis was extended for 4 weeks postoperatively. Anticoagulant or antiaggregant prophylaxis did not continue beyond this time point solely because of vein reconstruction.^16^

### Specimen Analysis

The specimens were assessed according to a standardized protocol based on axial slicing and circumferential margin evaluation. Each axial slice was examined on a single large slide measuring 45 × 40 mm^12^.

### Statistics

Categorical variables are summarized as frequencies, percentages, and rates. Continuous variables are expressed as mean ± SD if normally distributed, or as median and interquartile range (IQR) if not.

The Pearson *χ*^2^ test and Fisher exact test (if group population was <5) were used to compare categorical variables between different groups. The Cochran–Armitage test for trend and the Wilcoxon/Kruskal–Wallis test were used to compare ordinal and continuous variables, respectively.

Propensity score analysis was performed using a greedy nearest-neighbor 1-to-1 (caliper = 0.25) matching algorithm (R package MatchIt) to balance the possible confounders (age, sex, BMI, ASA score, diabetes, history of heart failure and pulmonary obstructive disease, and neoadjuvant chemotherapy) between the 2 groups.

For statistical significance of the test, a power of 80%, *P* < 0.05, the 2-tailed significance level was used.

All statistical analyses were performed using the JMP Pro 16.0.0 software package for Mac (Copyright SAS Institute Inc., SAS Campus Drive, Cary, NC) and R Package, R Core Team (2014): A language and Environment for Statistical Computing (R Foundation for Statistical Computing, Vienna, AT) version 4.3.0 (April 21, 2023).

## RESULTS

During the study period, 665 PD were performed, including 151 PD-VR (22.7%). Open and robotic PD-VR were performed in 115 (76.2%) and 36 (23.08%) patients, respectively. In robotic PD-VR 1 patient required elective conversion to open surgery (2.9%). The conversion rate for all robotic PD performed during the study period was 0.75% (2/267). In both patients, conversion to open surgery was required because of oozing bleeding, which was tedious to manage robotically.

In 151 patients with PD-VR, SPC were recorded in 35 patients (23.2%), leading to a postoperative mortality of 6.0%. SPC occurred in 7 patients after robotic PD-VR (19.4%) and in 28 patients after open PD-VR (24.4%) (*P* = 0.543; OR: 0.75 [0.30–1.90]). The equivalent figures for mortality were 8.3% and 5.2% (*P* = 0.446; OR: 1.65 [0.39–6.97]). Regarding the other outcome variables, robotic PD-VR was associated with longer mean operative time (610.0 ± 17.2 minutes vs 537.0 ± 9.6 minutes; *P* < 0.0001), more frequent use of type 4 vein resection and reconstruction (27.8% vs 9.6%; *P* = 0.0059), and a higher rate of PPH (27.8% vs 13%; *P* = 0.038). A trend toward statistical significance was noted for a shorter median length of hospital stay (15.5 [11.3–22.0] days vs 20.0 [14.0–27.0] days; *P* = 0.0736) in the robotic group. The histology of resected specimens showed that robotic PD-VR had a shorter median length of proven vein infiltration (8 [5–10] mm vs 10 [5–17.5] mm; *P* = 0.0195). The quality of histology is shown by the median number of examined lymph nodes (robotic PD-VR: 43.5 [34.3–56]) (open PD-VR: 44.0 [33.0–59.0]) (Supplemental Table 1, see http://links.lww.com/AOSO/A313).

### Comparison of Matched Cohorts

Before matching, patients undergoing robotic PD-VR had a lower median age (66.0 [55.5–71.0] years vs 70.9 [62.8–76.0] years; *P* = 0.0022) and lower ASA score (PD-VR: 2.5 [2.0–3.0] vs 3.0 [2.0–3.0]; *P* = 0.0454).

**FIGURE 1.**
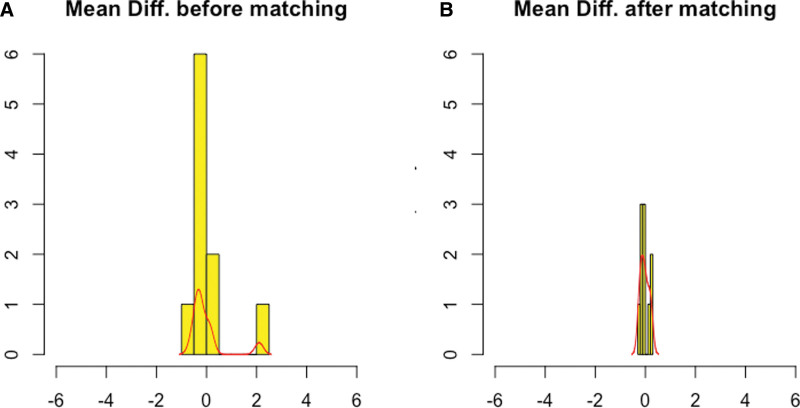
Histograms with overlaid kernel density estimates for standardized mean differences before (A) and after (B) propensity score matching, demonstrating reduction of imbalance for all covariates between the 2 groups.

The matching process identified 2 groups of 35 patients (Table 1). No differences in the preoperative characteristics were observed between the 2 groups. The standardized mean difference and mean empirical distribution function decreased from 2.1031 to 0.252, respectively, before matching to 0.127 and 0.0151, respectively, after matching (Fig. 1).

**TABLE 1.** Matching Parameters Before and After Propensity Score Matching

	Before Propensity Score Matching				After Propensity Score Matching			
	PD-VR	Open PD-VR	Robotic PD-VR	*P*	PD-VR	Open PD-VR	Robotic PD-VR	*P*
Patients	151	115 (76.2%)	36 (23.8%)		70	35 (50.0%)	35 (50.0%)	
Age; median (IQR), years	69.4 (61.5–75.0)	70.9 (62.8–76.0)	66.0 (55.5–71.0)	0.0022	66.0 (59.8–71.0)	64.5 (61.2–74.0)	66.0 (57.0–71.0)	0.503
Male gender; n (%)	85.0 (56.3%)	67.0 (58.3%)	18.0 (50.0%)	0.383	40.0 (57.1%)	22.0 (62.9%)	18.0 (51.4%)	0.334
Body mass index; mean ± SD, kg/m^2^	24.4 ± 3.2	24.4 ± 3.4	24.3 ± 2.5	0.774	24.3 ± 3.2	24.5 ± 3.9	24.2 ± 2.5	0.681
ASA score; median (IQR)	3.0 (2.0–3.0)	3.0 (2.0–3.0)	2.5 (2.0–3.0)	0.0454	2.5 (2–3)	2.0 (2–3)	3.0 (2–3)	0.638
Diabetes; n (%)	36 (23.8%)	26 (22.6%)	10 (27.8%)	0.525	21 (30%)	11 (31.4%)	10 (28.6%)	0.794
Heart failure; n (%)	32 (21.2%)	27 (23.5%)	5 (13.9%)	0.251	13 (18.6%)	8 (22.9%)	5 (14.3%)	0.540
Chronic obstructive pulmonary disease; n (%)	9 (6%)	9 (7.8%)	0 (0%)	0.115	0 (0%)	0 (0%)	0 (0%)	NA
Neoadjuvant chemotherapy; n (%)	22 (14.6%)	19 (16.5%)	3 (8.3%)	0.224	4 (5.7%)	1 (2.9%)	3 (8.6%)	0.614

The outcomes after propensity score matching are shown in Supplemental Table 2, see http://links.lww.com/AOSO/A313. SPC were recorded in 7 patients (20.0%) in the robotic group and in 6 patients (17.1%) in the open group (*P* = 0.759; OR: 1.21 [0.36–4.04]). Three patients died after robotic PD-VR (8.6%) and none died after open PD-VR (*P* = 0.239, OR: 97,338,565 [NA–NA]).

Robotic PD-VR was associated with longer operative time (611.1 ± 13.9 minutes vs 529.0 ± 13.0 minutes; *P* < 0.0001), more frequent use of type 2 (28.6% vs 5.7%; *P* = 0.0234), less frequent use of type 3 vein resection and reconstruction (31.4% vs 71.4%; *P* = 0.0008), longer vein occlusion time (30 [25.3–78.3] minutes vs 15 [8–19.5] minutes; *P* = 0.0098), less estimated blood loss (450 [200–750] mL vs 733 [500–1070.3] mL; *P* = 0.0075), and fewer intraoperative (14.3% vs 48.6%; *P* = 0.0041) and perioperative blood transfusions (14.3% vs 60.0%; *P* = 0.0001). One patient in either group (2.9%) developed partial thrombosis of the superior superior mesenteric/portal vein, which was treated with a therapeutic heparin drip.

Robotic PD-VR did not increase R1 rates in patients with PDAC. This result was achieved by comparing 2 groups of patients with similar T and N stages, the proportion of patients with proven vein infiltration, and the length of the vein infiltration.

## DISCUSSION

To the best of our knowledge, only one previous study has provided a propensity-matched comparison between minimally invasive and open PD-VR. Matched cohorts included 13 patients each. The minimally invasive group included 6 robotic PD-VR. Four of the remaining 7 PD had an open reconstruction and 3 were converted to open surgery during the resection phase. The minimally invasive group included 2 patients who underwent arterial resection.^8^ A few other studies have compared open and robotic PD-VR, but none in the context of propensity match analysis.^9,10^ Therefore, the current study provides the first matched comparison between open and robotic PD-VR.

Although the need for vein resection and reconstruction often suggests a borderline tumor, it is essential to note that these 2 conditions are not synonymous. Furthermore, borderline pancreatic tumors may also necessitate arterial resection. Although arterial resection may be feasible under robotic assistance,^16,18^ it adds complexity to an already intricate procedure. Therefore, the need for arterial resection was an exclusion criterion in this study, which aims to address specifically the issue of vein resection and reconstruction during robotic PD, rather than the broader field of robotic surgery for borderline resectable pancreatic tumors.

Robotic PD-VR was noninferior to open PD-VR with respect to the incidence of SPC. No differences were noted in the secondary study endpoints. Although the absence of improvement in clinical outcomes could be seen as a negative result, for an approach that was developed to curtail surgical morbidity, it is important that robotic PD-VR did not worsen outcomes in the context of a highly morbid operation, such as PD-VR. Concerning safety, it is also important that in PD-VR, there was only one elective conversion to open surgery. Vascular resection is a leading reason for conversion in robotic PD,^33^ especially if a segmental resection is required.^34^ Although elective conversion is not expected to have a negative impact on clinical outcomes, emergency conversion, which is often required to fix major bleeding, has been associated with increased rates of postoperative complications.^33^ Robotic assistance reduced the conversion rate.^35^ Our study shows that, in select patients, a careful approach permits the implementation of robotic PD-VR.

The main endpoint of this study was the incidence of SPC. In PD, SPC was associated with higher mortality,^36^ lower probability of receive adjuvant chemotherapy,^37^ and decreased overall and disease-free survival.^38^ SPC has also been shown to prolong the length of hospital stay and increase costs.^39^ In a recent multicenter study that provided a difficulty score for robotic PD (PD-ROBOSCORE), SPC was the main endpoint. In this score, 2 of the 5 difficulty parameters have clear implications for PD-VR (ie, borderline resectable pancreatic tumor and tumor located in the uncinate process of the pancreas).^40^ Therefore, it is important to note that robotic PD-VR did not increase the incidence of SPC compared to open PD-VR.

This study also provides additional information. Robotic PD-VR reduced the amount of blood loss and, therefore, the need for blood transfusions and did not compromise margin status. Blood transfusion affects both postoperative and oncological results.^41^ Therefore, having shown that robotic PD-VR decreases the risk of receiving blood transfusions is important piece of information.

Margin status is an important prognostic factor in resected PDAC and is a quality marker for surgical resection. This study showed that robotic PD-VR did not compromise the margin status. In addition, robotic PD-VR had low rates of portal vein thrombosis (2.9%). In a recent study, portal vein thrombosis and margin status were the main prognostic factors in a large series of patients with open PD-VR.^42^ It is important to note that most of our resections, both open and robotic, were R1. This result is in agreement with the concept that “most pancreatic cancer resections are R1 resections,” which was first proposed by Esposito et al,^43^ and raises concerns about the variability of margin assessment.^44^ The high R1 rates reported in this study can be explained by careful histology of resected specimens, but can also reflect the rare use of neoadjuvant therapies. More frequent use of neoadjuvant therapies will probably result in improved R0 rates. However, the guideline paradigm “borderline resectable pancreatic cancer = neoadjuvant therapy” does not apply fully to clinical practice. In a large single-center series of open PD-VR, only 103 of 694 patients received neoadjuvant therapy (14.8%).^42^ In a multicenter study on PD-VR, only 414 of 1260 patients (32.9%) received preoperative treatments.^45^ In a benchmark study on PD-VR, only 28% of the patients received neoadjuvant chemotherapy.^5^ Although the importance of neoadjuvant therapy in PDAC is not under discussion,^46^ practice appears to be somewhat different from the theory.

Robotic PD-VR was associated with longer operative time and required some adaptations in the technique of vein resection when compared to open PD-VR.

It is now clear that robotic PD, and not specifically PD-VR, increases the operative time by approximately 100 minutes.^10,47^ A longer operative time could have an impact on clinical outcomes. In a recent study, the operative times for minimally invasive and open PD were divided into quartiles. For each quartile, the impact of operative time on overall morbidity was worse in open PD than in minimally invasive PD. However, in the upper quartile, a longer operative time leads to higher reoperation and mortality rates for minimally invasive PD.^48^ A study by the Pittsburgh group defined the learning curve of PD-VR based on the operative time. The analysis included 50 patients (type 1 resection: 43, 86.0%; type 2 resection: 6, 12.0%; type 3: 1, 2.0%). Operative time dropped after the first 8 procedures, plateaued between cases 8 and 35, and decreased again thereafter. A shorter operative time (444 vs 362 minutes) was associated with less blood loss (350 vs 200 mL) but did not improve morbidity and mortality. In that study, conversion to open surgery in PD-VR was required in 10% of operations. The rates of blood transfusions, SPC, and mortality were 22%, 28%, and 8%, respectively.^34^ These data compare favorably with those of the present study. Despite a higher level of complexity, as shown by the need for segmental vein resection in >60% of the patients, there was only one conversion, and the rates of blood transfusion, SPC, and mortality were 14%, 17%, and 8.6%, respectively. Although a reduction in operative time is important, we believe that the outcome of robotic PD-VR depends more on the meticulous surgical technique than on operative time. In robotic PD-VR, the operative time can be reduced if effective energy devices are available. The tip of the robotic vessel sealer was too bulky to permit precise dissection in deep and narrow spaces. Robotic harmonic shears are appropriate in size and shape, but cannot articulate and are associated with lateral thermal spread that raises concerns about the use of this device on the adventitial plane of large visceral arteries.^49^ A newer robotic synchroseal could be an improvement. The lack of robotic instruments that are fully suitable for retroperitoneal dissection has prompted several surgeons to proceed with laparoscopic dissection followed by robotic reconstruction.^50^ According to the Brescia guidelines, such an approach should be defined “roboscopic” rather than robotic. If laparoscopic energy devices are used to improve robotic dissection, the procedure should be identified as “robotic-assisted”. Finally, if only robotic instruments are used, the procedure should be defined as “pure robotic.”^7^ In this study robotic PD-VR was performed using a “pure robotic” technique. This approach may have contributed to the increased operative time.

In a recent study, we presented the technical reasons for adaptations in vein reconstruction in robotic versus open PD-VR. These adjustments are required because of the limited possibility of performing direct vascular reconstruction while maintaining a reverse Trendelenburg position, difficulties in achieving safe vascular control when the superior mesenteric vein is involved near the mesenteric root, and the need to manage the splenic vein when the spleno-portal junction is involved. Consequently, more type 2 and fewer type 3 vein resections were performed in the robotic PD-VR.^14^

This study had several limitations. First, although propensity score-matched analysis is considered the closest approximation to randomization, this study was based on a retrospective analysis. Therefore, it can carry all biases associated with this type of analysis. Second, even with propensity score matching, unmeasured and/or unknown confounders could not have been balanced between the 2 groups. However, we believe that the most important and clinically meaningful confounders were covered. Third, robotic PD-VR is technically demanding. The fact that a group of surgeons who pioneered these procedures in an open setting and were in charge of a transplant program was able to implement robotic PD-VR (in select patients) does not mean that this could be easily duplicated. Therefore, the generalizability of the results presented in this study needs to be established. Fourth, the need for vascular resection is among the major reasons for conversion from robotic surgery to open surgery. This study reported an exceedingly low rate of conversion to open surgery (0.75%), which is inconsistent with the literature. Notably, this result was not achieved by insisting on a robotic approach beyond the limits of safe practice, as shown by the low rate of blood transfusions. The low rate of conversion is an additional factor that raises concerns regarding the generalizability of the results presented herein. Fifth, because there were few patients who underwent neoadjuvant chemotherapy, this study was unable to address the crucial questions about the feasibility and safety of robotic PD-VR after these treatments. Nonetheless, the data presented in this study are important and unique at this time because at least 50% of PD-VR are unplanned procedures that are consequently carried out on patients who have not received neoadjuvant therapies. Finally, this study did not report data on intraoperative patient management by the anesthesia team. Intraoperative medical management is extremely important and may affect the incidence and severity of postoperative complications. However, this issue should be addressed in future studies.

This study had several strengths. First, each robotic PD-VR was performed upon completion of the learning curve in standard robotic PD. In addition, the operating surgeon (U.B.) had previous experience with robotic renal and pancreatic transplants, ensuring the acquisition of skills in vascular anastomosis before robotic PD-VR. Second, this study compared contemporary open and robotic PD-VR performed by the same surgeon (U.B.), managed by the same anesthesia team, and overseen by the same surgical team. Therefore, group differences should only be related to surgical approaches. Third, because the robot was not always timely available, some of the patients who received open PD-VR were actually selected for robotic PD. This aspect enhances comparability between the control and study groups, even if randomization was not used.

In conclusion, this noninferiority propensity-matched study showed that robotic PD-VR did not increase the risk of SPC. The value of robotic PD-VR needs to be further addressed in randomized controlled trials involving surgeons specifically trained to overcome the technical challenges of this formidable operation.

## Supplementary Material


