# Supplementary material for: Robotic Versus Open Pancreatoduodenectomy With Vein Resection and Reconstruction: A Propensity Score-Matched Analysis
Source: Ann Surg Open. 2024 Mar 26;5(2):e409. doi: 10.1097/AS9.0000000000000409 (PMC11191888; doi:10.1097/AS9.0000000000000409)
Supplement: Supplementary file 1 [file as9-5-e409-s001.pdf]

Supplemental table 1. Operative, postoperative and histology data in open and robotic PD-VR.

	**PD-VR** (n= 151)		**Open PD-VR** (n= 115)	**Robotic PD-VR** (n= 36)	**p**
**Operative data**					
Operative time; mean±SD, min	554.4±107.5		537±108.2	610±84.9	0.0003
Conversion; n (%)	1 (0.7%)		-	1 (2.9%)	NA
Vein resection; n (%)					
Type 1	12 (7.9%)		8 (6.9%)	4 (11.1%)	0.481
Type 2	13 (8.6%)		3 (2.6%)	10 (27.8%)	<0.0001
Type 3	105 (69.5%)		93 (80.9%)	12 (33.3%)	<0.0001
Type 4	21 (13.9%)		11 (9.6%)	10 (27.8%)	0.0059
Estimated blood loss; median (IQR), mL	600 (301.6-1030)		674.1 (360-1095)	435 (210.4-746.1)	0.0108
Intraoperative blood transfusions; n (%)	57 (37.7%)		52 (45.2%)	5 (13.9%)	0.0007
Perioperative blood transfusions; n (%)	74 (49%)		69 (60%)	5 (13.9%)	<0.0001
Vein occlusion time; median (IQR), min	22 (15-30)		15 (10-18.8)	30 (25.3-78.3)	0.0030
Pylorus preserving; n (%)	115 (76.2%)		87 (75.7%)	28 (77.8%)	0.794
Pancreatojejunostomy; n (%)	146 (96.7%)		110 (95.7%)	36 (100%)	0.339
**Postoperative data**					
Initial intensive care stay; median (IQR), days	1 (1-3)		1 (1-3)	1 (1-2)	0.0545
Return to intensive care; n (%)	14 (9.3%)		11 (9.6%)	3 (8.3%)	1.000
Overall intensive care stay; median (IQR), days	1 (1-3)		2 (1-3)	1 (1-2)	0.0736
Hospital stay; median (IQR), days	19 (13-26)		20 (14-27)	15.5 (11.3-22)	0.0697
Functional recovery; median (IQR), days	3 (3-6)		3 (3-5)	3 (3-10.5)	0.587
Textbook outcome; n (%)	102 (68.9%)		77 (68.1%)	25 (71.4%)	0.714
Severe postoperative complications; n (%)	35 (23.2%)		28 (24.4%)	7 (19.4%)	0.543
Grade IIIa	12 (7.9%)		11 (9.6%)	1 (2.9%)	0.295
Grade IIIb	8 (5.3%)		5 (4.4%)	3 (8.3%)	0.397
Grade IVa	5 (3.3%)		5 (4.4%)	0	0.339
Grade IVb	1 (0.7%)		1 (0.9%)	0	1.000
Grade V	9 (6.0%)		6 (5.2%)	3 (8.3%)	0.446

CR-POPF; n (%)	15 (9.9%)		14 (12.2%)	1 (2.9%)	0.121
Grade C	3 (2.0%)		2 (1.7%)	1 (2.9%)	0.561
PPH (grade B/C); n (%)	25 (16.6%)		15 (13%)	10 (27.8%)	0.038
DGE (grade B/C); n (%)	32 (21.2%)		21 (18.3%)	11 (30.6%)	0.115
Bile leak; n (%)	4 (2.7%)		2 (1.8%)	2 (5.6%)	0.221
Chyle leak; n (%)	2 (1.3%)		1 (0.9%)	1 (2.9%)	0.421
Vein thrombosis; n (%)	2 (1.3%)		1 (0.9%)	1 (2.9%)	0.426
Interventional radiology procedures; n (%)	16 (10.6%)		14 (12.2%9	2 (5.6%)	0.360
Interventional endoscopy procedures; n (%)	1 (0.7%)		1 (0.9%)	0	1.000
Repeat surgery; n (%)	16 (10.6%)		10 (8.7%)	6 (16.7%)	0.175
90-day readmission; n (%)	6 (3.9%)		3 (2.6%)	3 (8.3%)	0.147
**Histology data***					
Pancreatic adenocarcinoma; n (%)	119 (78.8%)		87 (75.7%)	32 (88.9%)	0.0899
Tumor size; median (IQR), mm	30 (25-35)		30 (25-35)	30 (25.5-37.8)	0.894
T stage; n (%)					
T1	12 (10.1%)		9 (10.3%)	3 (9.4%)	1.000
T2	90 (75.6%)		66 (75.9%)	24 (75%)	0.923
T3	17 (14.3%)		12 (13.8%)	5 (15.6%)	0.774
T4	0		0	0	NA
Examined lymph nodes; median (IQR)	44 (33-58)		44 (33-59)	43.5 (34.3-56)	0.472
Metastatic lymph nodes; median (IQR)	3 (1-7)		3 (1-6)	2.5 (1.3-7)	0.871
N stage; n (%)					
N0	18 (15.1%)		14 (16.1%9	4 (12.5%)	0.777
N1	45 (37.8%)		31 (35.6%)	14 (43.8%)	0.418
N2	56 (47.1%)		42 (48.3%)	14 (43.8%)	0.684
R0 margin; n (%)	59 (49.6%)		46 (52.9%)	13 (40.6%)	0.236
R1 margin; n (%)	60 (50.4%)		41 (47.1%)	19 (59.4%)	0.236
1. Anterior	15 (12.6%)		9 (10.3%)	6 (18.8%)	0.221
2. Posterior	28 (23.5%)		21 (24.1%)	7 (21.9%)	0.796
3. Vein	31 (26.1%)		18 (20.7%)	13 (40.6%)	0.0280
4. SMA	20 (16.8%)		14 (16.1%)	6 (18.8%)	0.731
5. Neck of the pancreas	1 (0.8%)		1 (1.2%)	0	1.000
6. Common bile duct	0		0	0	NA
7. Proximal duodenum	0		0	0	NA

≥2 R1 margins; n (%)	25 (21.0%)		15 (17.2%)	10 (31.3%)	0.0963
Vein infiltration; n (%)	61 (51.3%)		43 (49.4%)	18 (56.3%)	0.509
Length of vein infiltration; median (IQR), mm	10 (5-15)		10 (5-17.5)	8 (5-10)	0.0195
Adjuvant chemotherapy; n (%)	77 (51%)		55 (47.8%)	22 (61.1%)	0.164
Time from surgery to chemotherapy; median (IQR), days	80.5 (61.5-92.5)		81.5 (60.5-92)	79 (62.5-99.5)	0.940
Full course chemotherapy; n (%)	46 (59.7%)		29 (52.7%)	17 (77.3%)	0.0711

Min: minutes; **mm**: millimeters; **SMA:** Superior mesenteric artery.
* considered only PDAC

Supplemental table 2. Operative, postoperative and histology data in open and robotic PD-VR after propensity score matching

	**All matched PD** (n= 70)		**Open PD-VR** (n= 35)	**Robotic PD-VR** (n= 35)	**p**
**Main study endpoint**					
Severe postoperative complications; n (%)	12 (18.6%)		6 (17.1%)	7 (20.0%)	0.759
Grade IIIa	3 (4.3%)		2 (5.7%)	1 (2.9%)	1.000
Grade IIIb	4 (5.7%)		1 (2.9%)	3 (8.6%)	0.614
Grade IVa	3 (4.3%)		3 (8.6%)	0	0.239
Grade IVb	0		0	0	NA
Grade V	3 (4.3%)		0	3 (8.6%)	0.239
**Secondary study endpoints**					
Postoperative mortality; n (%)	3 (4.3%)		0	3 (8.6%)	0.239
Functional recovery; median (IQR), days	3 (3-7)		3 (3-4)	3 (3-11)	0.627
Textbook outcome; n (%)	50 (73.5%)		26 (76.5%)	24 (70.6%)	0.583
Hospital stay; median (IQR), days	18 (14-26)		19 (14-28)	16 (12-22)	0.143
CR-POPF; n (%)	6 (8.6%)		5 (14.3%)	1 (2.9%)	0.198
Grade C POPF; n (%)	1 (1.4%)		0	1 (2.9%)	1.000
PPH (grade B/C); n (%)	16 (22.9%)		6 (17.1%)	10 (28.6%)	0.255
DGE (grade B/C); n (%)	17 (24.3%)		6 (17.1%)	11 (31.4%)	0.163
Repeat surgery; n (%)	8 (11.4%)		2 (5.7%)	6 (17.1%)	0.260
90-day readmission; n (%)	5 (7.1%)		2 (5.7%)	3 (8.6%)	1.000
R0 resection; n (%)	25 (44.6%)		13 (52.0%)	12 (38.7%)	0.320
Examined lymph nodes*****; median (IQR)	45 (34.5-58.8)		45 (35.5-60.5)	45 (34-56)	0.438
**Reasons for repeat surgery**					
Hemorrhage ; n (%)	6 (8.6%)		1 (2.9%)	5 (14.3%)	0.198
Bowel obstruction; n (%)	1 (1.4%)		0	1 (2.9%)	1.000
Wound dehiscence; n (%)	1 (1.4%)		1 (2.9%)	0	1.000
**Reasons for hospital readmission**					
Abdominal fluid collections; n (%)	2 (2.9%)		2 (5.7%)	0	0.493
Vomit; n (%)	1 (1.4%)		0	1 (2.9%)	1.000
Ascites; n (%)	1 (1.4%)		0	1 (2.9%)	1.000

Hepatic artery pseudoaneurysm; n (%)	1 (1.4%)		0	1 (2.9%)	1.000
**Other outcome measures**					
Operative time; mean±SD, min	570.2±91.5		529.3±78.6	611.1±85.9	<0.0001
Conversion; n (%)	1 (1.4%)		-	1 (2.9%)	NA
Vein resection; n (%)					
Type 1	7 (10%)		3 (8.6%)	4 (11.4%)	1.000
Type 2	12 (17.1%)		2 (5.7%)	10 (28.6%)	0.0234
Type 3	36 (51.4%)		25 (71.4%)	11 (31.4%)	0.0008
Type 4	15 (21.4%)		5 (14.3%)	10 (28.6%)	0.244
Vein occlusion time; median (IQR), min	25 (15-36.5)		15 (8-19.5)	30 (25.3-78.3)	0.0098
Estimated blood loss; median (IQR), mL	600 (301.3-961.2)		733 (500-1070.3)	450 (200-750)	0.0075
Intraoperative blood transfusions; n (%)	22 (31.4%)		17 (48.6%)	5 (14.3%)	0.0041
Perioperative blood transfusions; n (%)	26 (37.1%)		21 (60.0%)	5 (14.3%)	0.0001
Pylorus preserving; n (%)	52 (74.3%)		25 (71.4%)	27 (77.1%)	0.584
Pancreatojejunostomy; n (%)	69 (98.6%)		34 (97.1%)	35 (100%)	1.000
Initial intensive care stay; median (IQR), days	1 (1-2)		1 (1-3)	1 (1-2)	0.4015
Return to intensive care; n (%)	6 (8.6%)		3 (8.6%)	3 (8.6%)	1.000
Overall intensive care stay; median (IQR), days	1 (1-2)		1 (1-3)	1 (1-2)	0.424
Functional recovery; median (IQR), days	3 (3-7)		3 (3-4)	3 (3-11)	0.627
Bile leak; n (%)	3 (4.4%)		1 (2.9%)	2 (5.7%)	1.00
Chyle leak; n (%)	1 (1.4%)		0	1 (2.9%)	1.00
Vein thrombosis; n (%)	2 (2.9%)		1 (2.9%)	1 (2.9%)	1.00
Interventional radiology procedures; n (%)	6 (8.6%)		4 (11.4%)	2 (5.7%)	0.673
Interventional endoscopy procedures; n (%)	1 (1.4%)		1 (2.9%)	0	1.000
Pancreatic adenocarcinoma; n (%)	56 (80.0%)		25 (71.4%)	31 (88.6%)	0.0730
Tumor size*****; median (IQR), mm	30 (25-35)		30 (25-35)	30 (25-38)	0.683
T stage*****; n (%)					
T1	6 (10.7%)		3 (12.0%)	3 (9.7%)	1.000

T2	42 (75%)		19 (76.0%)	23 (74.2%)	0.877
T3	8 (14.3%)		3 (12.0%)	5 (16.1%)	0.720
T4	0		0	0	NA
Positive lymph nodes*****; median (IQR)	3.5 (1-7)		4 (1-7)	2 (1-7)	0.778
N stage*****; n (%)					
N0	7 (12.5%)		3 (12%)	4 (12.9%)	1.000
N1	21 (37.5%)		8 (32%)	13 (41.9%)	0.580
N2	28 (50%)		14 (56%)	14 (45.2%)	0.420
R1 margin*****; n (%)	31 (55.4%)		12 (48%)	19 (61.3%)	0.320
Anterior margin	9 (16.1%)		3 (12.0%)	6 (19.4%)	0.716
Posterior margin	12 (21.4%)		5 (20.0%)	7 (22.6%)	1.000
Vein margin	18 (31.1%)		5 (20.0%)	13 (41.9%)	0.0940
SMA margin	11 (19.6%)		5 (20.0%)	6 (19.4%)	1.000
Pancreatic neck margin	0		0	0	NA
Common bile duct margin	0		0	0	NA
Proximal duodenal margin	0		0	0	NA
≥2 R1 margins*****; n (%)	14 (25%)		4 (16%)	10 (32.3%)	0.220
Vein infiltration*****; n (%)	26 (46.4%)		9 (36.0%)	17 (54.8%)	0.160
Length of vein infiltration*****; median (IQR), mm	8 (5-10)		8.5 (5.3-10)	8 (5-10)	0.675
Adjuvant chemotherapy; n (%)	43 (61.4%)		22 (62.9%)	21 (60%)	0.806
Time from surgery to chemotherapy; median (IQR), days	80 (58-92)		81 (52.5 – 92)	79 (62.5 – 99.5)	1.000
Full course chemotherapy; n (%)	32 (74.4%)		16 (72.7%)	16 (76.2%)	1.000

Min: minutes; **mm**: millimeters; **SMA:** Superior mesenteric artery.
* Histology data refer to patients with pancreatic adenocarcinoma
